# To mask or not to mask: Debunking the myths of mask-wearing during COVID-19 across cultures

**DOI:** 10.1371/journal.pone.0270160

**Published:** 2022-09-29

**Authors:** Rong Chen, Bih-Jen Fwu, Tong-Rong Yang, Yi-Kai Chen, Quang-Anh Ngo Tran

**Affiliations:** 1 Department of Psychological and Brain Sciences, Colgate University, Hamilton, New York, United States of America; 2 Center for Teacher Education, National Taiwan University, Taipei, Taiwan; 3 Department of Psychology, National Taiwan University, Taipei, Taiwan; VIT University, INDIA

## Abstract

Mask-wearing is the simplest yet most effective preventive behavior during COVID-19. However, it has sparked great controversy, particularly in America. Little is known about what psychosocial factors predict people’s decision to mask. This research challenges three myths about mask-wearing. First, does mask-wearing provide a false sense of security? Second, is knowledge of COVID-19 a more robust predictor than political ideology of mask-wearing behavior? Third, does resistance to masks reflect anti-authoritarianism or a lack of trust in government? With nationally representative samples across two cultures (*N* = 1,121), findings reveal a significant positive correlation between mask-wearing and other preventive behaviors. Moreover, knowledge of COVID-19 and trust in government significantly predicted mask-wearing. Implications of the results are also discussed in the cross-cultural context. Critically, findings could provide practical implications for public education and policymaking by uncovering how to more effectively promote compliance with recommended preventive behaviors during our ongoing struggle with COVID-19.

## Introduction

What predicts people’s mask-wearing behavior during a pandemic? As a precaution to COVID-19, mask-wearing seems to be a simple preventive behavior. Indeed, universal mask-wearing has been recommended to limit the transmission of the novel coronavirus [1, 2]. Despite some initial hesitancy, public health officials now see masks as a powerful weapon against the virus, particularly after the World Health Organization acknowledged that the virus can be airborne [3], with tiny respiratory droplets able to linger in the air for hours. The director of the CDC in July 2020 stated that if all Americans had embraced rigorous mask-wearing, the country could have controlled the virus within one to two months [4].

Nevertheless, mask-wearing has sparked great controversy (especially in the early phases of the pandemic). In the U.S., in particular, it is not merely an issue of public health but also a political one. Even over a year after the virus was first detected, resistance to masks still lingers. For example, the Federal Aviation Administration stated that airlines have reported 1,900 incidents of unruly passengers refusing to wear face masks [5]. Given the evidence of its efficacy to reduce the transmission of the novel coronavirus, why did such a clash of science and politics emerge and persist? Little is known about what psychosocial factors might predict people’s decision to mask.

### Myth #1: Mask provides a false sense of security

This research is exploratory in nature and aims to debunk three myths about mask-wearing to prevent the transmission of COVID-19. First, there are concerns that mask-wearing could engender a false sense of security in relation to other methods of infection control such as social distancing and handwashing. However, there is little empirical evidence to support the contention that wearing masks would mean other approaches to infection control would be overlooked [1]. On the contrary, a study conducted between April and May 2020 with a German sample reveals that mask-wearing correlated positively with other protective behaviors [6]. Does mask-wearing provide a false sense of security? We hypothesized that there will be a significant positive correlation between wearing masks and other approaches to infection control, i.e., handwashing and social distancing (H1).

### Myth #2: Ideology is the main driver of mask use

Second, the politics of mask-wearing has been coined the new culture war in the U.S. [7]. There is some evidence that the debate is split along party lines—partisanship was found to be a fairly strong predictor of one’s likelihood of wearing a mask [8–11]. This finding is in line with partisan differences regarding other COVID-19 precautionary measures such as physical distancing [12, 13]. Nonetheless, polls show that 70–80% of Americans have worn a mask in public [14]. Previous findings in East Asia reveal that knowledge is key—having correct knowledge of the pandemic was linked to increased preventive behavior. For instance, preventive practice was enabled by knowledge of the causes of the 2009 H1N1 influenza pandemic [15].

Furthermore, during the avian influenza (AI) outbreak in East Asia, people who had correct knowledge about AI were more likely to practice AI preventive behavior [16]. Greater knowledge of AI (e.g., knowing correctly the modes of AI transmission and proper AI preventive measures) was associated with increased odds of adopting preventive measures, including wearing protective clothing and face masks. Specifically, compared with participants who misperceived the fatality rate of AI vs. H1N1, those with correct knowledge about AI were more than four times as likely to practice the recommended AI preventive behavior [16]. Does the new culture war really exist in the U.S.? Or is knowledge of COVID-19 a more robust predictor of people’s mask-wearing behavior?

### Myth #3: It is all about personal freedom

Third, one commonly cited resistance to masks is personal freedom. People do not want the government telling them what to do. Mask requirements have thus been considered a serious infringement or a threat to personal freedom. Escalating tension over the precaution has spurred protests, fights, and even a fatal shooting [17]. This perceived government overreach could be related to anti-authoritarian tendency [18], since authoritarianism denotes a tendency to submit willingly to strong authority, as opposed to supporting individual freedom and responsibility. Notwithstanding, the authority’s guidance has been inconsistent (particularly in the initial phases of the pandemic). During much of the COVID-19 pandemic in 2020, the U.S. government lacked a clear direction [19]. Indeed, recommendations on masks varied greatly between countries and dynamically changed over time [20]. There also seems to be an inconsistency between people’s behavior and attitude—polling indicates that people wear masks while believing the decision to wear a mask should be a personal choice [14]. How can we resolve such a paradox?

The most recent pandemic recorded prior to COVID-19 was the H1N1 influenza outbreak in 2009. Previous research with H1N1 responses revealed that trust in government was strongly associated with adherence to health guidelines [21–23]. The public will have to trust experts and officials before it cooperates with their recommendations. It was found that trust in government agencies such as the Ministry of Health was related to an increase in all the recommended behaviors for the 2009 H1N1 influenza pandemic among Italians [22]. Additionally, in a two-wave longitudinal survey of adults in Switzerland, trust in medical organizations such as the WHO predicted perceived efficacy of officially recommended protection measures, including wearing a mask [21]. No other variables explained significant amounts of variance. Indeed, following the AI and H1N1 pandemics, commentators have speculated on the deleterious impact that a crisis of trust between the public and health authorities could have on compliance with recommendations in the case of future pandemics [24].

In short, trust in government plays a crucial role in shaping people’s preventive behavior; the public does not necessarily trust the government’s recommendations and that lack of trust will impact the extent of its cooperation. Does resistance to masks reflect people’s anti-authoritarianism or rather a lack of trust in government? Building on previous findings reviewed above, we hypothesized that trust in government and knowledge of COVID-19 will make the strongest uniquely significant contribution to the explanation of mask-wearing behavior (H2).

### Other cultural contexts

Beyond the U.S., masks have been broadly accepted in many European and Asian countries. The main aim of this research was to examine the topic in a cross-cultural context, in order to further validate the proposed hypotheses. As such, the initial focus was to understand the key predictors of mask use across cultures, above and beyond various sociocultural variables, as opposed to cross-cultural comparison per se.

Interestingly, while there is thought to be a mask-wearing culture in East Asia, it is argued to be rather an impact of the SARS outbreak in 2003 [25]. In Taiwan, for example, the government learned from its 2003 SARS experience and established a public health response mechanism for enabling rapid actions for the next crisis. Consequently, COVID-19 infection and death rates have been low despite its proximity to mainland China. In particular, the government took an active role in educating the public in addition to resource allocation, including daily briefings to the public. Bloomberg ranked Taiwan #3 in the global COVID Resilience Ranking [26] and a Brookings Institution study named Taiwan #1 for COVID response [27]. As such, Taiwan is deemed an exemplar of how a democratic society can respond quickly to a crisis and protect the interests of its citizens [28, 29].

This research represents an important topic of current research as mask-wearing is the visual consequence of a pandemic that is present in the everyday lives of most people. Individual decisions made during the COVID-19 pandemic shape the course of the virus’s spread and the risks facing human populations. Critically, findings could provide practical implications for public education and policymaking by uncovering how to more effectively promote compliance with recommended preventive behaviors during our ongoing struggle with COVID-19.

## The present research

This research aims to debunk three myths about mask-wearing: (a) Mask-wearing provides a false sense of security, (b) Political ideology is the main predictor of people’s mask-wearing behavior, (c) It is all about personal freedom. Building on previous findings, our hypotheses were two-fold: (a) There will be a significant positive correlation between wearing masks and other approaches to infection control, i.e., handwashing and social distancing (H1), and (b) Knowledge of COVID-19 and trust in government will make the strongest uniquely significant contribution to the explanation of mask-wearing behavior (H2). Both hypotheses along with the study design, planned sample size, inclusion/exclusion criteria, and planned primary analyses were pre-registered on aspredicted.org (https://aspredicted.org/blind.php?x=jd8jz9). We examined what predicts mask-wearing in a representative Western culture, the U.S., by testing the proposed hypotheses with a nationally representative sample of American adults in late 2020. In addition, we investigated mask-wearing behavior in a benchmark case in East Asia, Taiwan, by testing the hypotheses with a nationally representative sample of Taiwanese adults in early 2021. Across both samples, we also sought to control for various psychosocial variables previously suggested to correlate with adherence to public health measures, such as perceived threat of COVID-19 [30].

## Method

This research was approved by the Institutional Review Board of Colgate University (protocol ER-F20-06). Each participant gave written consent before taking part online.

### Participants

#### U.S. sample

A nationally representative sample of 560 American adults in terms of age, gender, race, and ethnicity was recruited via Cloud Research for this study (i.e., quotas based on race, gender, age, and ethnicity, matched to 2010 census estimates, were included to ensure a demographically representative sample of the U.S. adult population). Each participant was compensated USD $2 for their participation. A priori power analysis using G*Power [31] indicated that a sample of 267 is needed to ensure adequate power (1-β ≥ .80) to detect a small/medium effect (*f*^*2*^ = .07). Twenty-four participants were excluded for failing to consent or complete the survey. Five participants were also excluded for failing the attention check questions, resulting in a valid sample size of 531 (45.88% male, 53.72% female, and 0.4% other). Participants’ age ranged from 18 to 88 (*M*_*age*_ = 46.2, *SD* = 17.91). For a snapshot of the sample characteristics, please refer to Table 1.

**Table 1:** Demographic characteristics of American and Taiwanese participants.

Variable	U.S.	Taiwan
	% of Sample	% of Sample
Race		
White	72.78	
Black or African American	11.49	
Mixed Raced	5.65	
Hispanic or Latino	4.64	
Asian	3.63	
Native American or Native Alaskan	1.01	
Native Hawaiian or Pacific Islander	0.20	
Han		94.70
Indigenous		1.65
New Immigrants		0.37
Other races or prefer not to answer	0.60	3.29
Marital Status		
Married	44.87	56.67
Single	44.47	40.04
Other	10.66	3.29
Residence		
West Coast	17.10	
Midwest	22.13	
Northeast	26.36	
South	34.41	26.87
North		47.53
Central		21.94
Other		3.66
Education		
Less than high school	3.42	2.01
High school graduate	24.55	16.09
Some college	20.32	
2-year degree	9.05	
4-year degree	24.55	
University		64.35
Master’s degree	12.47	16.27
Doctorate	3.82	1.28
Professional degree	1.81	
Annual Income		
Less than USD$10,000	10.26	
$10,000 - $19,999	9.46	
$20,000 - $29,999	13.48	
$30,000 - $39,999	10.87	
$40,000 - $49,999	10.06	
$50,000 - $59,999	7.65	
$60,000 - $69,999	4.43	
$70,000 - $79,999	6.24	
$80,000 - $89,999	2.62	
$90,000 - $99,999	5.23	
$100,000 - $149,999	12.47	
More than $150,000	7.24	
Monthly Income		
Less than NTD$10,000		13.53
$10,000 - $19,999		7.13
$20,000 - $29,999		16.27
$30,000 - $39,999		20.48
$40,000 - $49,999		14.26
$50,000 - $59,999		11.15
$60,000 - $69,999		5.67
$70,000 - $79,999		4.57
$80,000 - $89,999		2.38
$90,000 - $99,999		1.83
$100,000 - $149,999		1.65
More than $150,000		1.1

*Note*. Table reflects participants who chose to report their demographic information (*n* = 497 in the U.S., *n* = 547 in Taiwan).

#### Taiwan sample

A nationally representative sample of 561 Taiwanese adults in terms of age, gender, and residence were recruited via a local market research agency for this study (i.e., quotas based on gender, age, and residence, matched to 2010 census estimates, were included to ensure a demographically and geographically representative sample of the Taiwanses adult population). Each participant was compensated TWD $60 (approximately USD $2) for their participation. To ensure data quality, fourteen participants whose responses demonstrated response sets (e.g., answering *strongly agree* to all survey questions) were excluded, resulting in a valid sample size of 547 (47.9% male, 50.27% female, and 1.83% other). Participants’ age ranged from 21 to 81 (*M*_age_ = 47.78, *SD* = 15.8).

### Measures

All materials and data for this research are available at the Open Science Framework website: osf.io/7nwuy. The survey was originally constructed in English and then translated into Mandarin by first-language-speaking authors, with subsequent independent back-translation and correction based on discussion [32].

#### Mask-wearing behavior

To measure compliance behavior, participants were asked to self-report the frequency objectively rather than subjectively (e.g., “I often wear a mask”). Specifically, three COVID-19 compliance behaviors were measured, including the frequency of mask-wearing, handwashing and social distancing. Participants responded by self-reporting the frequency of each behavior ranging from 0% to 100%. In particular, two items used to measure mask-wearing behavior read, “I wear a mask ___ of the time when around people indoors” and “I wear a mask ____ of the time when around people outdoors.” Higher scores indicate higher frequency of mask-wearing and other COVID-19 compliance behaviors.

#### COVID-19 perceived threat

The 3-item short version of the Perceived Coronavirus Threat Questionnaire [30] was used to measure perceived threat of COVID-19 (α = .86 for the U.S. sample, α = .78 for Taiwan sample). Participants responded on a five-point Likert scale ranging from 1 (*strongly disagree*) to 5 (*strongly agree*). A sample item from the scale read, “Thinking about the coronavirus makes me feel threatened.” Higher scores indicate higher individual perceived threat of the COVID-19 pandemic.

#### COVID-19 perceived reality

Items were adapted from Conway et al. (2020), participants were asked to indicate whether they have known someone infected with or died of COVID-19 and whether they have been tested for COVID-19 (U.S. sample: α = .48, Taiwan sample: α = .75) [30]. Participants responded using a six-point Likert scale ranging from 1 (*strongly disagree*) to 6 (*strongly agree*). Higher scores indicate higher individual perception of the reality of the COVID-19 pandemic.

#### Authoritarianism

We adopted the 6-item Very Short Authoritarianism (VSA) scale [33] to measure authoritarian tendency (U.S. sample: α = .59, Taiwan sample: α = .63). Participants responded using a five-point Likert scale ranging from 1 (*strongly disagree*) to 5 (*strongly agree*). A sample item from the scale read, “What our country needs most is discipline, with everyone following our leaders in unity.” Higher scores indicate higher authoritarian tendency.

#### Political ideology

Participants were asked to self-identify their own political ideology on a scale ranging from 1 (*extremely liberal*) to 5 (*extremely conservative*), with higher scores representing more conservative orientation. For validation in the U.S. sample, we used chi-square independence test to assess the association of self-report ideology with participants’ 2020 Presidential vote choice (Biden, Trump, or Other) and partisan affiliation (Democrat, Republican, Other, or None). Results indicated that ideology had large effects associated with presidential vote choice (*χ*^2^ = 215.78, *df* = 12, *p* = .001, Cramér’s $\hat{V}$ = .38) and partisan affiliation (*χ*^2^ = 134.63, *df* = 8, *p* = .001, Cramér’s $\hat{V}$ = .37). Participants who identified as “Conservative” voted for Trump 65.3% and affiliated with the Republican Party 64.7%, while participants who identified as “Liberal” voted for Biden 85.8% and affiliated with the Democratic Party 82.1%. In short, those who voted and identified with Democrat in our sample are relatively liberal and those who identified with and voted Republican are relatively conservative.

To fit the Taiwanese context, we validated self-report ideology using chi-square independence test to assess the association of self-report ideology with participants’ 2020 Presidential vote choice (Tsai, Han, Soong, or Not applicable) and partisan affiliation (DPP, Democratic Progressive Party; KMT, Kuomintang; or Other). Results indicated that ideology had medium effects associated with presidential vote choice (*χ*^2^ = 53.97, *df* = 12, *p* = .001, Cramér’s $\hat{V}$ = .18) and partisan affiliation (*χ*^2^ = 56.76, *df* = 8, *p* = .001, Cramér’s $\hat{V}$ = .23). Participants who identified as “Conservative” voted for Han 31.7% and affiliated with KMT 41.3%, while participants who identified as “Liberal” voted for Tsai 59.5% and affiliated with DPP 31.3%. In short, those who identified with the DPP and voted for the incumbent, Ying Wen Tsai, in our sample are relatively liberal and those who identified with the KMT and voted for its candidate, Guo Yu Han, are relatively conservative.

#### Knowledge of COVID-19

To measure knowledge of the pandemic and prevention of the transmission of COVID-19, we used both a 5-item self-reported subjective knowledge scale (e.g., “I am well informed on the issue of COVID-19”) and a 6-item objective knowledge scale (e.g., “Masks can block dangerous respiratory droplets that would otherwise be spread by infected people”). Participants responded using a six-point Likert scale ranging from 1 (*strongly disagree*) to 6 (*strongly agree*). We then calculated a composite “knowledge of COVID-19” score to be used in subsequent analyses, with higher scores representing higher knowledge of the COVID-19 pandemic (U.S. sample: α = .74, Taiwan sample: α = .72).

#### Trust in government

To measure trust in government, we asked participants to respond to 5 items assessing their trust in the government generally (e.g., institutions such as the CDC, α = .75) in terms of percentage (0–100%) as well as their trust in the government’s response to the pandemic specifically (e.g., “I think the authorities are well prepared for the COVID-19 outbreak,” U.S. sample: α = .78, Taiwan sample: α = .85) on a six-point Likert scale ranging from 1 (*strongly disagree*) to 6 (*strongly agree*). Items assessing general trust in government were modified (e.g., institutions such as the CDC in Taiwan, α = .83) to fit the Taiwanese context. Higher scores indicate higher trust in the American/Taiwanese government.

#### SARS experience

Given Taiwan’s previous SARS experience, we also included measures to assess its impact on people’s mask use during COVID-19. Three items were included to assess prior experience with SARS: “Prior experience with SARS taught me how to better take preventive measures,” “I trust the government’s guidance based on its SARS experience,” and “SARS showed that we must combat a pandemic with unity.” Participants responded on a six-point Likert scale ranging from 1 (*strongly disagree*) to 6 (*strongly agree*).

#### Demographic control variables

Participants self-reported their age, gender (dummy coded with males serving as the reference category), race (dummy coded with Asian as the reference category), marital status (dummy coded with married as the reference category), residence (dummy coded with West Coast as the reference category), educational attainment, and annual household income.

For the Taiwanese sample, the race (dummy coded with Han serving as the reference category) and residence (dummy coded with North as the reference) options were modified; participants were asked to report their monthly income in New Taiwanese Dollar (NTD).

### Procedure

After giving their written consent at the beginning, participants responded to the questions outlined above in an online survey. All questions were randomized. They also answered the demographic questions before reading the debrief form and signing off the webpage.

## Results

### U.S.

Since data collection took place amid the pandemic and our survey included questions asking whether participants have known someone died of COVID-19, participants were granted the right to skip any questions they did not feel comfortable answering. To handle missing data, we used the full information maximum likelihood (FIML) method and followed Newman’s (2014) guidelines [34]. Of the 531 participants, 435 (81.7%) answered all items, 54 (10%) answered at least one item of each construct (item-level missingness), and 42 (8.3%) answered at least one construct (construct-level missingness). For item-level missingness, we used available items to represent the construct. For construct-level missingness, we used the FIML method to estimate hierarchical regression models through PROC CALIS and pairwise deletion method to conduct other statistical analyses. All data analyses were performed in SAS version 9.4.

To validate H1, we conducted multivariate regression to examine the effect of mask-wearing in predicting handwashing and social distancing (controlling for sociopolitical variables). MANOVA was first performed to examine whether mask-wearing can explain the covariance matrix between handwashing and social distancing. Results indicated that Wilks’ Lambda = 0.803 (*F*_(2, 482)_ = 59.24, *p* < .001 under α = .05), suggesting that mask-wearing significantly predicted the covariance matrix between social distancing and handwashing. Next, as reported in Table 2, controlling for sociopolitical variables, mask-wearing positively predicted both social distancing and handwashing, indicating that mask-wearing is highly related to social distancing and handwashing. This supports our first hypothesis, debunking the myth that wearing masks provides a false sense of security by overlooking other approaches to infection control.

**Table 2:** Results of regression analysis for US and TW data.

	US sample								TW sample							
Predictor variable	Social distancing				Handwashing				Social distancing				Handwashing			
	β	SE	*t*		β	SE	*t*		β	SE	*t*		β	SE	*t*	
Authoritarianism	-.90	1.23	-.74		-.61	1.35	-.45		3.73	1.40	2.66	**	-.43	1.63	-.26	
Political ideology	-1.87	.77	-2.44	**	-1.29	.85	-1.52		.36	.77	.46		1.03	.90	1.14	
General rust in government	.30	.05	6.31	**	.20	.05	3.74	**	.05	.06	.88		-.03	.07	-.46	
Specific trust in government	.67	1.00	.67		-3.50	1.10	-3.18	**	-.66	1.32	-.50		3.09	1.55	2.00	*
Mask-wearing	.32	.04	8.99	**	.29	.04	7.47	**	.27	.04	7.58	**	.43	.04	10.24	**
*R* ^*2*^	.29				.19				.14				.18			

*Note*.

* *p* < .05

** *p* < .01.

To validate H2, we conducted a three-stage hierarchical multiple regression analysis. We entered all demographic variables and the psychosocial variables (COVID-19 perceived threat and reality) at stage one (S1); authoritarianism and political ideology were entered at stage two (S2); knowledge of COVID-19 and trust in government were entered at stage three (S3). Descriptive statistics and correlation matrix among key variables are displayed in Tables 3 and 4.

**Table 3:** Descriptive statistics for U.S. and Taiwan samples.

	*M*		*SD*		*Minimum*		*Maximum*	
Variable	U.S.	Taiwan	U.S.	Taiwan	U.S.	Taiwan	U.S.	Taiwan
Mask-wearing	71.90	76.05	25.85	23.08	0.00	0.00	100.00	100.00
COVID-19 perceived threat	3.60	3.99	1.14	0.84	1.00	1.00	5.00	5.00
COVID-19 perceived reality	3.33	1.11	1.44	0.31	1.00	1.00	6.00	3.00
Authoritarianism	3.17	3.37	0.76	0.60	1.00	1.40	5.00	5.00
Political ideology	2.97	2.29	1.20	1.07	1.00	1.00	5.00	5.00
Knowledge of COVID-19	4.92	4.69	0.64	0.52	2.36	1.42	6.00	6.00
General trust in government	63.54	66.05	20.85	19.94	0.00	0.00	100.00	100.00
Specific trust in government	3.63	4.37	0.99	0.88	1.57	1.71	6.00	6.00

**Table 4:** Bivariate correlations among key variables.

Taiwan																
Taiwan→	Mask wearing		Perceived threat		Perceived reality		Authoritarianism		Political ideology		Knowledge of COVID-19		General trust in government		Specific trust in government	
U.S.↓																
Mask-wearing	—		.27	***	-.07		.23	***	-.04		.28	***	.18	***	.10	*
Perceived threat	.38	***	—		.02		.18	***	.04		.20	***	.05		.06	
Perceived reality	.11	*	.19	***	—		.04		.12	**	-.05		.04		-.03	
Authoritarianism	-.06		.00		-.05		—		.15	***	.25	***	.18	***	.10	*
Political ideology	-.10	*	-.19	***	-.06		.29	***	—		-.06		-.10	*	-.18	***
Knowledge of COVID-19	.34	***	.27	***	.04		.01		-.02		—		.30	***	.35	***
General trust in government	.27	***	.33	***	.08		.13	**	-.16	***	.36	***	—		.72	***
Specific trust in government	-.04		.17	***	.11	*	.29	***	.11	*	-.02		.37	***	—	
U.S.																

Note.

* *p* < .05

** *p* < .01

*** *p* < .001. U.S. data presented below the diagonal; Taiwan data presented above the diagonal.

As shown in Table 5, hierarchical multiple regression revealed that at S1, the demographic and psychosocial variables accounted for 20% of the variation in the dependent variable and contributed a medium effect [35] to the regression model. Introducing S2 explained an additional 1% of the variation in the dependent variable (controlling for the effects of variables at S1), although the overall R^2^ (20%) remained less than a small effect. Adding S3 to the regression model explained an additional 8% of the variation in mask-wearing and this change made the overall R^2^ (28%) reached a large effect, suggesting that knowledge of COVID-19 and trust in government contributed significantly to the S3 model. When all the independent variables were included in S3 of the regression model, the most significant predictors of mask-wearing were perceived threat of COVID-19 (standardized *β* = .286, *p* < .001), race (White, standardized *β* = -.210, *p* = .027), knowledge of COVID-19 (standardized *β* = .193, *p* < .001), general trust (standardized *β* = .169, *p* < .001) and specific trust in government (standardized *β* = -.118, *p* = .021).

**Table 5:** Hierarchical regression analysis of predictors of mask-wearing (U.S. sample).

	Stage 1				Stage 2				Stage 3			
Predictor Variable	β	SE	*t*		β	SE	*t*		β	SE	*t*	
Age	.05	.04	1.13		.06	.04	1.33		-.02	.05	-.41	
Gender female	.04	.04	1.04		.05	.04	1.07		.02	.04	.53	
Gender non-binary	-.04	.04	-1.10		-.05	.04	-1.10		-.04	.04	-.92	
White	-.25	.10	-2.53	*	-.25	.10	-2.49	*	-.21	.10	-2.21	*
Black or African American	-.16	.08	-1.97	*	-.15	.08	-1.88		-.11	.08	-1.48	
Mixed Raced	.00	.06	.02		.00	.06	.03		.02	.06	.33	
Hispanic or Latino	-.09	.06	-1.45		-.09	.06	-1.45		-.08	.06	-1.44	
Other races	.04	.04	.80		.04	.04	.80		.04	.04	1.06	
Native American or Native Alaskan	-.02	.05	-.53		-.02	.05	-.53		-.02	.04	-.53	
Native Hawaiian or Pacific Islander	.01	.04	.12		.01	.04	.14		.02	.04	.44	
Marital status single	.06	.05	1.09		.05	.05	.96		.04	.05	.85	
Marital status other	.07	.05	1.58		.07	.05	1.43		.04	.04	.95	
Residence Midwest	.09	.06	1.55		.09	.06	1.57		.08	.05	1.53	
Residence Northeast	.06	.06	1.03		.06	.06	1.10		.05	.05	.97	
Residence South	.10	.06	1.64		.10	.06	1.66		.08	.06	1.50	
Education	.05	.05	1.01		.05	.05	.89		.03	.05	.71	
Income	.00	.06	.03		.00	.06	.00		.00	.05	-.04	
Perceived threat	.37	.04	9.09	***	.37	.04	9.04	***	.29	.04	6.74	***
Perceived reality	.05	.04	1.16		.05	.04	1.14		.04	.04	.92	
Authoritarianism					-.05	.04	-1.21		-.03	.04	-.79	
Political ideology					.01	.05	.15		.03	.04	.68	
Knowledge of COVID-19									.19	.04	4.55	***
General trust in government									.17	.05	3.53	***
Specific trust in government									-.12	.05	-2.30	*
*R* ^*2*^	.20				.20				.28			
*R*^*2*^ change					.00				.08			

Note.

* *p* < .05

*** *p* < .001. *R*^*2*^ was calculated by one minus the proportion of residual variance of mask-wearing to the total variance.

## Discussion

In line with previous research [6], results from the U.S. sample validated H1 and demonstrated that there is a significant positive correlation between mask-wearing and handwashing as well as social distancing during COVID-19. This debunks the myth that mask-wearing would provide a false sense of security by overlooking other preventive measures. Furthermore, the current finding supported H2, albeit to a lesser degree than expected. Controlling for a host of demographic and psychosocial variables, knowledge of COVID-19 and trust in government contributed significantly to predicting mask-wearing behavior, in addition to threat perception. This suggests that people will mask if they know why it is important. Instead of being driven by ideology, people’s trust in the government is crucial: generally, higher trust in various government agencies predicts people’s decision to adopt recommended preventive measures such as mask-wearing; specifically, people tend to take a more relaxed approach when trusting that the government is handling the pandemic well. This supports previous research suggesting that better government intervention reduces stress along with the perceived need for compliance, if things seem well in hand [36]. In the U.S. context, we also found that compared with Asian Americans, people who identified as White tended to mask less. Might there be a cultural explanation to this finding? We address this issue in the next step.

## Results

### Taiwan

To validate H1 and see if results from the U.S. sample could be replicated, we repeated the multivariate regression analyses on the COVID-19 compliance behaviors in the Taiwan sample. Results showed that Wilks’ Lambda = 0.806 (*F*_(2, 540)_ = 65.02, *p* < .001 under α = .05), suggesting that mask-wearing significantly predicted the covariance matrix between social distancing and handwashing. Next, controlling for sociopolitical variables, mask-wearing positively predicted both social distancing and handwashing (please see Table 2), indicating that mask-wearing is highly related to social distancing and handwashing. This replicated the results from the U.S. sample and supported our first hypothesis, debunking the myth that wearing masks would engender a false sense of security and mean that other preventive measures are overlooked.

To validate H2, we repeated the three-stage hierarchical multiple regression analysis. As shown in Table 6, hierarchical multiple regression revealed that at S1, the demographic and psychosocial variables accounted for 10% of the variation in the dependent variable and contributed a small to medium effect to the regression model. Introducing S2 explained an additional 3% of the variation in the dependent variable (controlling for the effects of variables at S1), and the overall R^2^ (14%) reached a medium effect. Adding S3 to the regression model explained an additional 5% of the variation in mask-wearing and this change made the overall R^2^ (19%) much greater than a medium effect, suggesting that knowledge of COVID-19 and trust in government contributed significantly to the S3 model. When all the independent variables were included in S3 of the regression model, results showed that SARS experience was not a significant predictor. The most significant predictors of mask-wearing were perceived threat of COVID-19 (standardized *β* = .209, *p* < .001), knowledge of COVID-19 (standardized *β* = .197, *p* < .001), general trust (standardized *β* = .182, *p* = .001) and specific trust in government (standardized *β* = -.138, *p* = .019), and authoritarianism (standardized *β* = .140, *p* = .001).

**Table 6:** Hierarchical regression analysis of predictors of mask-wearing (Taiwan sample).

	Stage 1				Stage 2				Stage 3			
Predictor Variable	β	SE	*t*		β	SE	*t*		β	SE	*t*	
Age	.04	.05	.79		-.01	.05	-.10		-.03	.05	-.59	
Gender female	.04	.04	.94		.03	.04	.76		.04	.04	.88	
Gender bisexual	.08	.04	2.02		.06	.04	1.54		.06	.04	1.44	
Gender other	-.05	.04	-1.17		-.05	.04	-1.31		-.05	.04	-1.35	
Gender prefer not to answer	.08	.04	1.98		.08	.04	1.85		.06	.04	1.48	
Race indigenous	.01	.04	.26		.02	.04	.40		.01	.04	.36	
Race new immigrants	.01	.04	.23		.02	.04	.47		.01	.04	.23	
Race other	.00	.04	-.01		.00	.04	-.09		.00	.04	.11	
Marital single	-.02	.05	-.40		-.02	.05	-.40		-.03	.05	-.53	
Marital other	.00	.04	.03		.01	.04	.20		.01	.04	.14	
Residence Central	.02	.05	.39		.03	.04	.58		.02	.04	.47	
Residence South	.02	.04	.46		.02	.04	.37		.01	.04	.33	
Residence East	-.08	.04	-1.85		-.07	.04	-1.79		-.07	.04	-1.79	
Education	.00	.05	.07		.02	.05	.44		.02	.04	.55	
Income	-.03	.05	-.66		-.01	.05	-.26		-.02	.04	-.50	
Perceived threat	.27	.04	6.70	***	.24	.04	5.95	***	.21	.04	5.18	***
Perceived reality	-.08	.04	-2.00		-.08	.04	-1.93		-.08	.04	-1.96	
Authoritarianism					.20	.04	4.56	***	.14	.04	3.17	**
Political ideology					-.07	.04	-1.68		-.05	.04	-1.21	
Knowledge of COVID-19									.20	.04	4.57	***
General trust in government									.18	.06	3.20	**
Specific trust in government									-.14	.06	-2.19	*
SARS experience									.00	.05	-.03	
*R* ^*2*^	.10				.14				.19			
*R*^*2*^ change	.00				.03				.05			

Note.

* *p* < .05

** *p* < .01

*** *p* < .001. *R*^*2*^ was calculated by one minus the proportion of residual variance of mask-wearing to the total variance.

## Discussion

By analyzing the dataset of a nationally representative sample in an East Asian cultural context, namely, Taiwan, we replicated results from the U.S. and provided evidence that people who adopt mask-wearing behavior also tend to social distance and wash hands frequently. Again, this finding supports H1 and debunks the myth that mask-wearing provides a false sense of security by overlooking other preventive measures. More importantly, results also showed that knowledge of COVID-19 and trust in government contributed significantly to mask-wearing behavior, replicating the pattern in the U.S. sample. This effect was robust to a host of control variables, including political ideology. In addition to threat perception, authoritarian tendency was found to significantly predict people’s decision to mask in the East Asian context. In short, our findings entail that while people’s general trust in government at the institutional level might be less likely to be built overnight, people’s specific trust in government including crisis management coupled with public education is also key in promoting adherence to guidance during a pandemic.

## General discussion

What are the most important predictors of mask-wearing? Across two nationally representative samples, we show that mask-wearing significantly correlated with handwashing and social distancing during the COVID-19 pandemic cross-culturally (controlling for sociopolitical variables). Furthermore, knowledge of COVID-19 and trust in government significantly predicted people’s decision to mask.

Findings from the U.S. sample revealed that people who wore masks also took other preventive measures (i.e., handwashing and social distancing) against COVID-19; moreover, higher knowledge and general trust in government predicted mask-wearing behavior. These patterns were replicated in Taiwan. The converging results underscore the robustness of the link between knowledge as well as trust and mask-wearing across cultures, debunking Myths #2 and #3, respectively. Crucially, this link was robust to a broad set of control variables, including political ideology. The current findings thus debunk the myth that people who mask overlook other preventive measures (Myth #1). More importantly, they debunk the myth that ideology predicts people’s decision to mask (particularly in the U.S. context).

Previous literature has identified several predictors of mask-wearing behavior, including perceptions of efficacy [11, 37], social norms [37, 38], psychosocial correlates [39, 40], and belief in science [11]. The present research adds to this line of literature by highlighting the importance of trust in government and knowledge in predicting mask-wearing. In line with previous findings [41, 42], our studies show that trust in government is key in predicting people’s COVID-19 compliance behavior. Also, the more people know, the better they behave. This corroborates previous finding that telling people to “rely on their reasoning” increases intentions to wear a face mask [39, 40], particularly if the message is more collectivistic (e.g., “good to your community”) [37].

It should be noted that perceived threat of COVID-19 predicted mask-wearing most strongly in both cultures. We speculate that since our data collection took place during the height of the pandemic (in December 2020 before the Christmas holiday and in February 2021 before the Chinese New Year holiday in the U.S. and Taiwan, respectively), this is understandable. Indeed, our results conform with existing COVID-19 research showing that more concerned individuals tend to display more behavioral compliance [11, 36]. For instance, Barceló and Sheen (2020) found that perceived threat (risk) predicted mask-wearing in Spain [38]. Interestingly, research has also found that empathy promotes the motivation to wear a face mask beyond vulnerability perceptions, suggesting that threat perceptions to the coronavirus are unlikely to be altered in an empathic state [43]. We posit a link between perceived threat and emotion on mask-wearing behavior which remains an intriguing topic for future research.

Comparing the findings across cultures, it is interesting to note that authoritarian tendency significantly predicted Taiwanese decision to mask consistently. This suggests that in cultures where the tendency to rely on authorities is valued, such tendency can translate into safe behaviors amidst uncertain times such as a global pandemic. This also underscores our finding that even in the U.S. context, those who identified as Asian Americans tended to mask more compared with Whites. In short, the present research reminds us of the effect of culture on human behavior, including how people respond to crises like the COVID-19 pandemic [44].

Altogether, our study extends the existing COVID-19 research by focusing on the simplest yet most effective preventive measure, mask-wearing, and examining psychosocial factors shaping its adoption. Furthermore, our study contributes to the cross-cultural psychological literature: on one hand, results highlight what motivates mask-wearing across cultures. On the other hand, results uncover why it is the most contentious preventive measure in some cultures while embraced unanimously in others.

It should be noted that the present research is rooted in contemporary public discourse. We hope this initial inquiry sheds light on the importance of the *why* (i.e., knowledge of mask-wearing, the more people know, the better they behave) and the *who* (i.e., trust in government) in predicting mask use and ultimately contributes to the development of a sound theory of mask-wearing behavior. We believe it is exactly this gap in research that makes the current study unique and valuable to extant literature.

### Limitations and future directions

Like all research, the current research has several limitations, providing opportunities for future research. First, given the risks of COVID-19, we relied on self-reported mask-wearing behavior rather than in-person observations. Future research could utilize the observational method or field experiments if possible. Second, while our research focused on two representative cases in the fight against COVID-19, future research should see if our results could be replicated in other cultural contexts. Third, future research could also examine how other psychological variables (e.g., emotion) might predict people’s mask-wearing behavior. Finally, as the pandemic progresses, whether and how the symbol of mask has changed (e.g., some people, especially those who have lost loved ones to coronavirus, may find it hard to let go of their masks, an item they have associated with saving lives for months) presents a fascinating topic for future research.

## Conclusion

As of early May 2021, more than 100 million Americans are fully vaccinated [45]. Notwithstanding, key questions remain about where and when to wear a mask–and this confusion is sparking political debates similar to ones seen in the early days of the pandemic. Individual decisions made during the COVID-19 pandemic shape the course of the virus’s spread and the risks facing human populations. We hope that findings from this research shed light on what predicts people’s mask-wearing behavior during a pandemic both within and outside of the U.S. context. As mask-wearing is the visual consequence of a pandemic that is present in the daily lives of most people, this research represents an important topic of current research. Critically, we hope that the present findings provide practical implications for public education and policymaking by uncovering how to more effectively promote compliance with recommended preventive behaviors during our ongoing struggle with COVID-19 across the globe.
